# Directing a colleague: LIKE EARLY STARVATION with FLOURY ENDOSPERM 6 targets isoamylase 1 to rice starch granules

**DOI:** 10.1093/plcell/koae070

**Published:** 2024-03-02

**Authors:** Nitin Uttam Kamble

**Affiliations:** Assistant Features Editor, The Plant Cell, American Society of Plant Biologists; John Innes Centre, Norwich Research Park, Norwich, NR4 7UH, UK

Starch is one of the most abundant biopolymers on Earth, constituting 80% to 90% of the rice grain endosperm weight, thereby greatly affecting crop yield and quality. In plants, starch is stored in the form of starch granules. Amylopectin and amylose polymers assemble in crystalline and amorphous regions of the starch granules. There are 3 classes of enzymes required for amylopectin biosynthesis: starch synthases, which elongate linear glucan chains; starch branching enzymes responsible for adding α-1,6 branch points; and the starch debranching enzymes named isoamylases (ISAs), which remove improper branch points. In addition to these enzymes, non-enzymatic proteins with starch-binding domains are emerging to be important for starch synthesis. An example is LIKE EARLY STARVATION (LESV), a widely conserved non-enzymatic protein with a C-terminal region rich in aromatic and acidic amino acid residues, originally discovered in Arabidopsis (Feike et al. 2016). Several roles have been proposed for LESV, including a role in the assembly of amylopectin helices into starch granules (Liu et al. 2023) and in modifying the surface structure of starch granules to influence enzymatic activities (Singh et al. 2022).

In this issue of *The Plant Cell*, **Haigang Yan, Wenwei Zhang, Yihua Wang, and colleagues** (Yan et al. 2024) identified a rice mutant, *floury endosperm9* (*flo9*), that is defective in starch accumulation from an N-methyl-N-nitrosourea–mutagenized mutant library. They showed that the *flo9* mutation was in the rice ortholog of LESV, providing an opportunity to investigate the role of LESV in the context of storage starch synthesis in grain, which differs significantly from leaf starch metabolism in Arabidopsis. Using immunoblot analysis, the authors showed that *Os*LESV exhibits a gradient increase from the exterior to the interior of the grain endosperm. The mutation in *Os*LESV resulted in grain endosperms with a translucent periphery, a floury-white intermediate region, and a hollow core region (Fig.) Mutant grain showed a decrease in total starch resulting from amylopectin variation but unaltered amylose and protein content relative to the wild type. The chain-length distribution of total glucans was altered in the mutant grain, exhibiting a pronounced increase in the central regions of grain endosperm. Following these observations, the authors studied starch granule initiation during rice grain development after flowering in peripheral, intermediate, and central regions of grains. They noted atypical amyloplasts in mutant grains without compound granules in the central regions, akin to what was observed in *flo6* mutants (Peng et al. 2014), with negligible effects on the peripheral region and loosely bound, tiny granules in intermediate regions. Localization studies of *Os*LESV1 in rice protoplasts with different combinations of domain truncations indicated that *Os*LESV1 is associated with starch granules. This was further confirmed through immunoblot analysis of subcellular fractionations and using in vitro glucan binding assays. Because the *Os*LESV1 mutant grains partially phenocopied *isa1* mutants, the authors investigated protein interactions and confirmed that *Os*LESV1 interacts with *Os*ISA1 through its N-terminal region (Fig.). The association of *Os*ISA1 with starch granules was largely compromised upon transient transformation in *flo9* protoplasts. The authors therefore proposed that LESV plays an important role in starch synthesis by interacting with other proteins, in particular associating with FLO6 to facilitate the access of ISA1 to starch granules.

**Figure.**
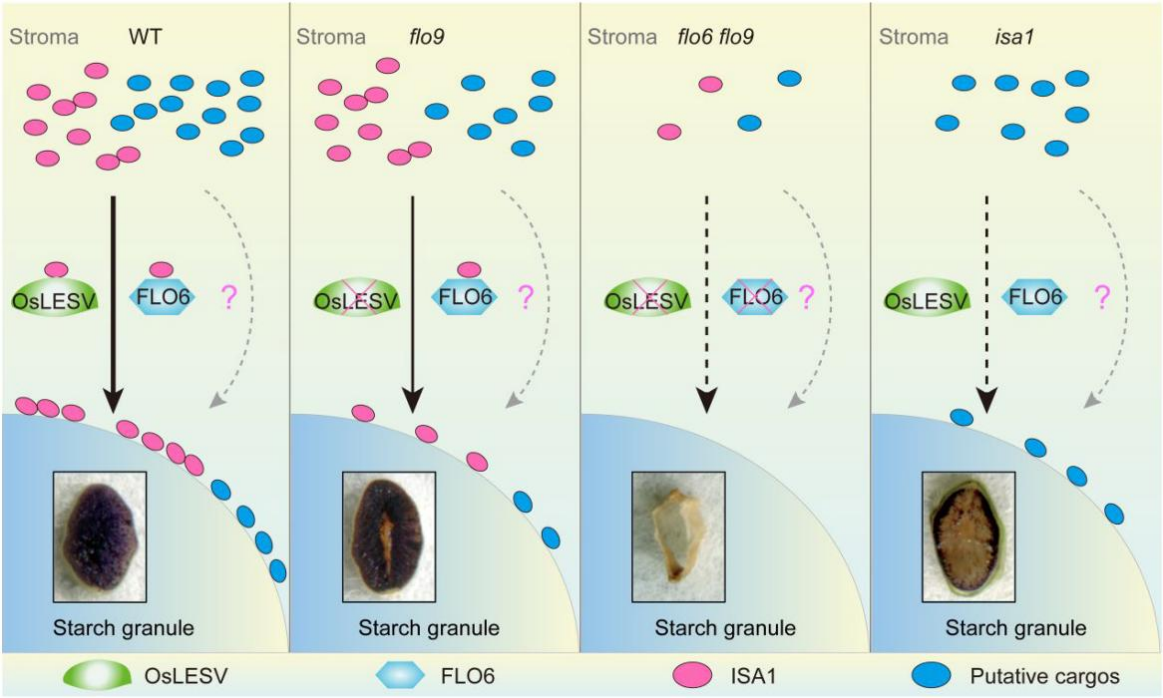
OsLESV associates with FLO6 to facilitate the access of ISA1 to starch granules in rice endosperm. OsLESV and FLO6 form a functional protein complex to recruit ISA1 from the stroma to starch granules to remove misplaced branches in amylopectin. Loss of any of these proteins drastically affects starch synthesis. Reprinted from Yan et al. (2024), Figure 7.

This mode of action represents a third possible mechanism of LESV function in starch synthesis, distinct from the previous 2 possibilities in amylopectin arrangement and surface structure modification from work in Arabidopsis (Singh et al. 2022; Liu et al. 2023). Although these 3 ideas differ, they are not mutually exclusive. The authors propose that further experiments could explore how these different hypotheses could be linked—for example, whether *Os*LESV can also affect the structure of glucans at the granule surface in a manner that enhances ISA1 binding. Additionally, it is also possible that LESV operates differently in different species/organs. Therefore, it will be important to identify other unknown cargos with which *Os*LESV associates in regulating starch metabolism.

## Dive Curated Terms

The following phenotypic, genotypic, and functional terms are of significance to the work described in this paper:

ISA1 Gramene: AT2G39930
ISA1 Araport: AT2G39930

amylopectin CHEBI: CHEBI:28057

starch CHEBI: CHEBI:28017
